# Sparse Blind Deconvolution Using ADMM Methods Based on Asymmetric Structured Prior for UWB Fuze

**DOI:** 10.3390/s25226986

**Published:** 2025-11-15

**Authors:** Shijun Hao, Xi Pan, Yanbin Liang, Kaiwei Wu, Bing Yang, Zhonghua Huang

**Affiliations:** School of Mechatronical Engineering, Beijing Institute of Technology, Beijing 100081, China; 3120215165@bit.edu.cn (S.H.); panxi@bit.edu.cn (X.P.); 3120195127@bit.edu.cn (Y.L.); 3120205163@bit.edu.cn (K.W.); 3120215164@bit.edu.cn (B.Y.)

**Keywords:** ultra-wideband (UWB) fuze, multipath propagation effects, ADMM, sparse blind deconvolution

## Abstract

The precise ranging of ultra-wideband (UWB) fuzes relies on extracting time delay information from echo signals. However, ground multipath propagation effects induce a significant time-delay spread in the echo signals. This manifests as a channel impulse response (CIR) composed of numerous, closely spaced components, creating a challenging super-resolution problem that severely constrains the ranging accuracy and reliability of the fuze. Therefore, accurately estimating the CIR that characterizes these multipath structures from a single echo observation is crucial for the UWB fuze to perceive terrain structures and enhance ranging capabilities. This study proposes the following methods: (1) establishing an equivalent discrete multipath model(EDMM) of the ground to characterize the CIR; (2) proposing a sparse blind deconvolution(SBD) method via the ADMM-based framework under an asymmetric structured prior (ASP), which employs parametric projections to constrain the physical morphology of the unknown source signal, and designing a periodic sparse cluster projection operator to achieve super-resolution recovery of the discrete multipath structure of the channel h by enforcing the EDMM prior. Through three-variable robust decomposition, it actively separates dispersed clutter and enhances performance under low signal-to-noise ratio (SNR) conditions. Experimental results from both simulations and measured data demonstrate that the proposed algorithm exhibits excellent robustness and recovery accuracy in complex low-SNR scenarios, providing a foundational offline analysis method for understanding complex channel characteristics and guiding the development of improved real-time ranging algorithms.

## 1. Introduction

Ultra-wideband (UWB) technology has been applied in various ground-proximity fuzes due to its high ranging accuracy and strong anti-interference capabilities [1,2]. The precise ranging of the UWB fuze relies on accurate measurement of the time of flight (ToF) of echo signals. However, when detecting extended, non-uniform complex targets such as the ground, multipath propagation effects inevitably spread a single pulse into a complex echo with temporal overlap. This temporal aliasing caused by delay spread obscures the time marker of the true distance by inducing peak ambiguity and energy center shift [3], thereby constraining the fuze’s ranging accuracy and reliability. Therefore, accurately recovering the underlying multipath structure—i.e., the channel impulse response (CIR)—that causes the delay spread, through deconvolution techniques, is crucial for enhancing the environmental awareness of the fuze and improving its operational effectiveness.

A variety of deconvolution approaches have been investigated to address this challenge. Among them, the CLEAN algorithm and its variants have been widely applied to UWB CIR measurements with sparse multipath characteristics, including in-vehicle [4,5], indoor and outdoor propagation [6,7], soil [8], and near-ground environments [9]. However, CLEAN still suffers from limitations in noise sensitivity and resolution [10], motivating the development of several alternative or complementary methods. For example, Lin [11] introduced a matching pursuit-based tap selection method to exploit the inherent sparsity of UWB channels for efficient equalization. Savelyev [12] developed a fast frequency-domain deconvolution algorithm for high-resolution UWB radar imaging. Hanssens [13] extended the RiMAX algorithm with an iterative maximum likelihood estimation scheme to achieve high-precision multipath parameter extraction. Rittiplang [14] proposed a sparse deconvolution approach with arctangent regularization, effectively suppressing noise amplification and enhancing imaging stability, and Nunoo [15] designed a sparsity-constrained LMS algorithm with iterative gradient updates for low-complexity, real-time estimation of time-varying UWB channels.

However, these deconvolution methods rely on prior knowledge of the UWB signal waveform. The true physical waveform of the source signal can undergo unknown distortion due to system non-idealities, and any prior mismatch may lead to severe distortion in the recovered results. This issue is particularly pronounced in the Equivalent Time Sampling (ETS) system employed in this study, as its time-scale transformation process further amplifies the uncertainty in the final equivalent source signal. It is this very uncertainty regarding the precise morphology of the source signal that renders supervised methods inadequate, thereby underscoring the necessity and importance of developing blind deconvolution techniques.

Blind deconvolution is a fundamentally underdetermined problem, as it seeks to recover both the source signal and the CIR from a single observation, yielding a solution space with extremely high degrees of freedom [16]. Incorporating sparsity priors effectively constrains the solution space and mitigates the inherent ambiguity [17]. Notably, UWB ground propagation channels inherently exhibit discrete multipath structures and sparsity, enabling near-unique recovery of the channel response even when the source signal is unknown. Therefore, sparsity-constrained blind deconvolution represents the most practical and robust approach for tackling such problems.

Sparse blind deconvolution (SBD) was originally developed for image deblurring and has since been widely extended to a variety of signal inversion problems [18]. In ultrasonic non-destructive testing (NDT), Gao [19] devised a two-phase algorithm featuring an initialization stage with blind gain calibration and ADMM, followed by an alternating optimization stage accelerated by PALM and MM methods, to recover severely overlapping and noisy echo signals. For ground-penetrating radar (GPR) data blind deconvolution, Jazayeri [20] proposed an SBD method that simultaneously estimates the source signal and subsurface reflectivity using an ℓ_2_-ℓ_1_ optimization scheme, without relying on prior knowledge of the wavelet. In the context of seismic data deconvolution, Repetti [21] introduced the Sparse Optimization via Optimal Thresholding (SOOT) algorithm, which integrates alternating minimization with forward-backward splitting, accelerated by a majorization-minimization (MM) framework. Within the Bayesian framework for sparse blind deconvolution, Civek [22] developed an improved estimation method based on a Normal-Inverse-Gamma (NIG) prior, solved efficiently using Markov Chain Monte Carlo (MCMC) techniques.

However, existing single-channel SBD frameworks still face three major challenges in non-cooperative scenarios:

(1) High sensitivity to initialization in a highly non-convex landscape;
(2) Lack of effective structured priors to resolve ambiguity in super-resolution regimes;
(3) Poor performance and instability under low SNR conditions.

Although strategies based on multi-channel observations [23] or known pilot signals [24] can alleviate these issues in specific applications, they are not applicable in the single-channel scenarios considered here. Minimum entropy deconvolution (MED) methods [25,26] primarily target source signal recovery and are insufficient for revealing the fine-grained structure of the CIR. Recent joint optimization approaches combining sparsity priors with dictionary learning [27] offer high modeling flexibility, but without structural guidance, they still rely on blind search in high-dimensional spaces and are prone to local minima. More advanced non-convex optimization frameworks have emerged, providing theoretical insights under specific assumptions. Li [28] established a landmark framework with provable convergence guarantees, but its reliance on random subspace assumptions limits its applicability to channels governed by structural physics. Wang [29] provided crucial theoretical understanding for L1-based methods, yet their core requirement of pulse separation directly contradicts the super-resolution challenge of overlapping echoes. The work of Guo and Bhandari [30] provides an elegant continuous domain solution by constructing source wavelets, but it relies on a Prony-like approach, making it only effective for low-order sparsity and not suitable for our dense multipath channel model. Therefore, there is an urgent need for a novel single-channel SBD framework that leverages domain-specific structural priors to ensure both reliable convergence and robustness in complex, low-SNR UWB conditions.

To address the aforementioned challenges of non-convexity, noise, and solution ambiguity, this paper proposes a two-stage blind deconvolution framework. First, the framework employs a data-driven strategy based on Singular Value Decomposition (SVD) for robust initialization, providing a high-quality starting point deep within the true solution’s basin of attraction for subsequent iterations. Second, within the core ADMM iterations, an innovative three-variable robust decomposition model is utilized to actively separate dispersed clutter. This model moves beyond the conventional two-variable paradigm by explicitly modeling structured echoes, non-periodic clutter, and noise, thereby enhancing the algorithm’s noise resistance. Most critically, under this purified framework, asymmetric and strongly structured priors are imposed to resolve the fundamental solution ambiguity and enable super-resolution. This prior breaks the problem’s symmetry by enforcing distinct physical constraints on the source signal s the channel response h: the physical morphology of s is strictly constrained via parametric projections, while a periodic sparse cluster projection operator is designed to precisely model the quasi-periodic structure of h. This channel-side prior is key to bypassing the coherence limitations inherent in separating severely overlapping echoes. Ultimately, this synergistic method can accurately recover the CIR characterizing ground multipath effects from a single echo, offering a novel technical pathway for UWB fuze to achieve environmental awareness and informed decision-making.

The remainder of this paper is structured as follows. Section 2 develops an equivalent discrete multipath model (EDMM) to characterize the ground CIR for UWB fuze. Section 3 formulates the blind deconvolution problem and introduces the initialization strategy along with the iterative optimization algorithm, incorporating the EDMM established in Section 2 as a prior constraint. Section 4 evaluates the performance of the proposed approach through comparative analysis against classical deconvolution methods. Finally, Section 5 concludes the paper and outlines potential future research directions.

## 2. UWB Impulse Response Model

**Figure 1:**
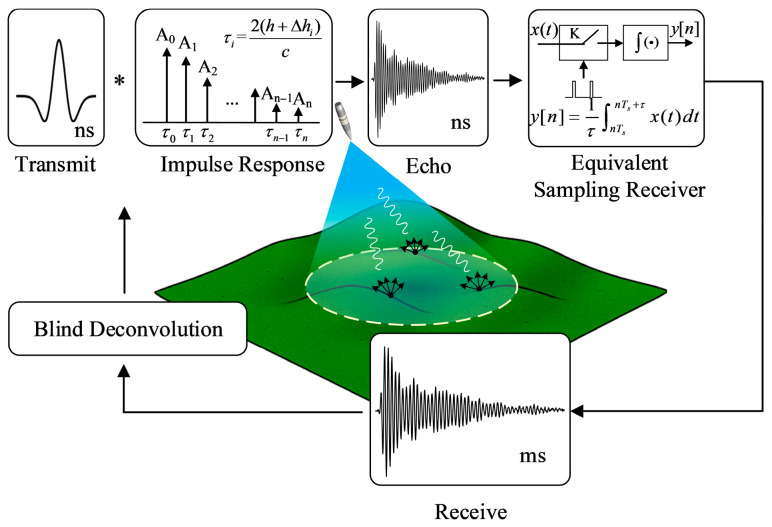
Framework of Blind Deconvolution for Ground Echo of UWB Fuze based on an Equivalent LTI System Model.

Figure 1 illustrates the core framework of this study, which performs blind deconvolution from the ground echo signal of UWB fuze. The forward physical model indicates that the equivalent source signal s, shaped by the system link and antenna transient response, is convolved with a physical channel h exhibiting delay spread due to ground multipath effects, resulting in a high-speed aliased echo x(t), which can be approximated by an equivalent linear time-invariant (LTI) model. This signal is then converted via ETS into the only known low- rate observed signal y[n]. The inverse problem is solved through blind deconvolution, where the blind nature of the problem arises from the unpredictability of s caused by dynamic coupling between the antenna and the near-field environment. Thus, the goal of the algorithm is to jointly recover the equivalent source signal $\hat{s}$ and the equivalent channel $\hat{h}$—which characterizes the ground multipath structure—using only the indirect equivalent observation y[n], even though both s, and h being unknown, thereby mitigating the ranging uncertainty induced by delay spread.

### 2.1. Ground Impulse Response Model

For typical terrain detected by UWB fuze, the surface is generally electromagnetically rough, and the most of its backscattered energy originates from incoherent diffuse reflection. This physical mechanism implies that each small rough unit illuminated by the antenna beam can be equivalently regarded as a secondary source radiating in all directions. Given that the distance between the fuze antenna and the ground is on the same order of magnitude as its illumination footprint on the ground, the wide beam angle inevitably receives backscattered echoes from ground scattering points at different distances simultaneously, resulting in geometric delay spread.

**Figure 2:**
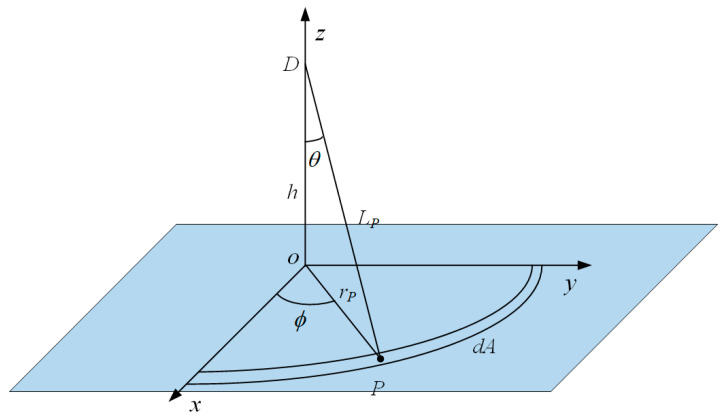
The geometric relationship between the ground scattering area and the fuze antenna.

Assume that the UWB fuze is located at (0, 0, h), and the scattering surface is a complex ground with random undulations, whose surface height is described by the function z = ζ(P), where P = (x,y) represents the horizontal coordinates (see Figure 2).

According to Huygens’ Principle and the radar equation, the channel response H(f) at frequency f results from the coherent superposition of echoes from all scattering points.

(1) $$H\left(f\right)=\int_{S}A\left(P\right)e^{-2\pi f\tau \left(P\right)}\cdot dS,$$

where A(P) is the complex amplitude associated with scattering point P, dS denotes the differential surface area, and τ(P) is the signal round-trip time delay.

Since the ground undulation height ζ being much smaller than the antenna height h, the path length L(P) can be decomposed into an average path length $L_{0}(r_{P})$ and a perturbation ΔL(P). The corresponding time delay is $\tau \left(P\right)\;=\;\tau_{0}\left(r_{P}\right)\;-\;\Delta \tau (P)$, where $r_{P}$ is the horizontal distance of the ground scattering point P.

By analyzing the ensemble average response of the channel and assuming that the scattering coefficient A is statistically independent of the surface undulation Δτ(P), and integrating the azimuth Angle ϕ, the result can be obtained.

(2) $$E\left[H(f)\right]=\int_{0}^{\infty }\left[2\pi \bar{A}(r_{P})C(r_{P},f)\right]\cdot r_{P}\cdot e^{-j2\pi f\tau_{0}\left(r_{P}\right)}dr_{P},$$

where $\bar{A}(r_{P})$ is the average scattering amplitude, $C\left(r_{P},f\right)\;=\;E\left[\exp(j2\pi f\Delta \tau )\right]$ is the coherence attenuation factor determined by the surface roughness.

Equation (2) indicates that the core phase structure $e^{-j2\pi f\tau_{0}(r_{P})}$ of the average channel response is exactly the same as that of an ideal flat ground. Therefore, the channel can be analyzed based on the ideal delay $\tau_{0}$.

Convert the integral variable from the spatial domain $r_{P}$ to the time-delay domain τ.

(3) $$r_{P}^{2}\left(\tau \right)=(\frac{c\tau }{2}{)}^{2}-h^{2},$$

where c is the propagation speed of electromagnetic waves. The contour with equal time delay forms a circle, and the area dS of its micro-element corresponds to the area $\left[\tau ,\;\;\tau \;+\;d\tau \right]$ of the ring in the interval.

(4) $$dS\left(\tau \right)=d\left(\pi r_{p}^{2}\right)=2\pi r_{P}dr_{P},$$

H(f) can be rewritten as

(5) $$H\left(f\right)\;={\;e}^{-j2\pi f\tau_{min}}\int_{0}^{\infty }g(\tau_{e})\cdot e^{-j2\pi f\tau_{e}}d\tau_{e},$$

where $\tau_{e}\;=\;\tau \;-\;\tau_{min}$ is the excess time delay; $\tau_{min}$ is the minimum propagation delay occurring at the reflection point $r_{P}\;=\;0$, and $\tau_{min}\;=\;2h/c$; $g(\tau_{e})$ includes the rate of change in amplitude and area.

(6) $$g\left(\tau_{e}\right)=A^{\prime }(\tau_{e}+\tau_{min})\left(\frac{\pi c^{2}(\tau_{e}+\tau_{min})}{2}\right),$$

Decompose the integration interval [$\left.0,\;\infty \right)$ of Equation (5) by period $T_{f}\;=\;1/f$.

(7) $$H\left(f\right)=e^{-j2\pi f\tau_{min}}\sum_{k=0}^{\infty }I_{k}(f),$$

where $I_{k}\left(f\right)\;=\;\int_{k/f}^{(k+1)/f}g(\tau_{e})e^{-j2\pi f\tau_{e}}d\tau_{e}$.

Within the interval $\left[k/f,(k+1)/f\right]$, perform the first-order Taylor expansion on $g(\tau_{e})$ with the midpoint $\tau_{k}^{*}\;=\;(k+1/2)/f$ as the center and substitute it into $I_{k}(f)$ to obtain

(8) $$I_{k}\left(f\right)\;\approx \;g(\tau_{k}^{*})\int_{k/f}^{(k+1)/f}e^{-j2\pi f\tau_{e}}d\tau_{e}+g^{\prime }(\tau_{k}^{*})\int_{k/f}^{(k+1)/f}(\tau_{e}-\tau_{k}^{*})e^{-j2\pi f\tau_{e}}d\tau_{e},$$

The integral of the zero-order term is zero, and the integral of the first-order term is calculated as $j/(\pi f^{2})$, thus

(9) $$I_{k}\left(f\right)\;\approx -\frac{c}{2(h+\frac{c(k+1/2)}{2f}{)}^{2}}\cdot \frac{j}{\pi f^{2}},$$

Equation (9) indicates that the channel’s response to a single frequency f is a coherent superposition of a series of contributing elements $I_{k}(f)$ arranged in a period $T_{f}$ in the time domain.

For a UWB signal with a central frequency of $f_{c}$, the average structure h(t) of its CIR can be efficiently approximated by an equivalent, time-equally spaced discrete multipath sequence $h_{d}(t)$.

(10) $$h_{d}\left(t\right)=\sum_{k=0}^{\infty }a_{k}\delta (t-t_{k}),$$

where $t_{k}$ is the position of each time window.

(11) $$\;t_{k}=\tau_{min}+kT_{c}=\frac{2h}{c}+\frac{k}{f_{c}},\;\;\;\;\;\;\;\;k=0,1,2\cdots N_{k},$$

The response intensity $a_{k}$ of $t_{k}$ at each moment is jointly determined by path loss, antenna directionality gain and ground scattering coefficient.

(12) $$a_{k}={\;C}_{h}\cdot G{\left(\theta (t_{k}),\phi (t_{k})\right)}^{2}\cdot \sqrt{\frac{\Gamma \left(\theta (t_{k})\right)}{(h+ct_{k}/2{)}^{3}}},$$

where $C_{h}$ is a constant term incorporating system-specific factors. $G\left(\theta (t_{k}),\phi (t_{k})\right)$ is the antenna power gain function, which is dependent on the incident angle θ and the azimuth angle ϕ. Both angles are functions of the time delay $t_{k}$. $\Gamma \left(\theta (t_{k})\right)$ is the ground backscattering coefficient.

Therefore, the expression of the equivalent discrete multipath model (EDMM) is

(13) $$h_{d}\left(t\right)=\sum_{k=0}^{\infty }\left[C_{h}G{\left(\theta (t),\phi (t)\right)}^{2}\sqrt{\frac{\Gamma \left(\theta (t)\right)}{{\left(h+ct/2\right)}^{3}}}\right]\cdot \delta \left(t-\left(\frac{2h}{c}+\frac{k}{f_{c}}\right)\right),$$

This model thus provides a parameterized skeleton for the ground impulse response, whose key parameters are directly governed by physical properties of the radar system and the terrain. The fundamental delay interval $T_{c}\;=\;1/f_{c}$ is dictated by the system’s center frequency, imprinting a periodic structure on the channel. The effective number of significant paths $N_{k}$ is determined by the interplay between the antenna beamwidth and the platform altitude h, which define the size of the illuminated area and thus the maximum delay of detectable scatterers. Crucially, the amplitude $a_{k}$ of each path, as specified in Equation (11), encapsulates the terrain-dependent physics. The ground backscattering coefficient $\Gamma \left(\theta \right)$ integrates the effects of two primary factors. The first is the complex dielectric constant, which governs the average reflection strength and its dependence on physical properties like soil moisture. The second is the surface microscopic roughness, which governs the transition from coherent to incoherent scattering and the associated random fluctuations in $\Gamma \left(\theta \right)$. This physically grounded interpretability of the EDMM parameters justifies the adoption of a strong, structured prior in the subsequent blind deconvolution algorithm.

### 2.2. UWB Signal Convolution Model

UWB signals are generated by narrow pulse generation circuits and can be represented in the form of the second derivative of a Gaussian pulse [31]. The mathematical expression is:

(14) $$s\left(t\right)\;=\;A_{p}\left[1\;-\;4\pi {\left(\frac{t}{\sigma }\right)}^{2}\right]e^{-2\pi {\left(\frac{t}{\sigma }\right)}^{2}},$$

where $A_{p}$ is the amplitude control coefficient of pulse signal; $\sigma \;=\;\Delta T/2\sqrt{\pi }$ is the width control coefficient of the pulse signal; ΔT is the pulse width.

While Equation (14) provides a specific model, a more general and powerful representation for such transient, time-localized, bandpass signals is the Gabor atom. In our framework, we adopt this parametric model for the unknown source signal s. This choice is justified by two fundamental reasons. Physically, a Gabor atom, being a Gaussian-windowed sinusoid, accurately captures the essential morphology of a typical UWB pulse. Mathematically, Gabor atoms are known to achieve the optimal joint time-frequency concentration dictated by the Uncertainty Principle, making them an extremely efficient and compact basis. This physically grounded parameterization constrains the unknown wavelet to a low-dimensional manifold, which is a crucial step for regularizing the ill-posed blind deconvolution problem.

Ideally, the received signal y of the UWB fuze can be modeled as a linear convolution of the unknown source signal s and the CIR h, which is interfered by noise n.

(15) y=s∗h+n,

where ∗ is the linear convolution operation.

However, in reality, the ground is a dissipative medium, and its scattering cross-section is a function of the incident angle as well as the frequency. During the scattering process of the source signal, there exists a problem of pulse shape distortion caused by the dispersion of the medium. To handle this issue reasonably within the framework of the classical LTI convolutional model, a key assumption is introduced: the waveform distortion caused by dispersion is equivalent to the superposition of several micro-multipath replicas of the original source signal wavelet.

Specifically, the pulse of the *i*-th path after dispersion distortion can be approximately represented as the convolution of the undistorted equivalent source signal and the distortion operator.

(16) $$s_{i}\left(t\right)=s(t)*d_{i}(t),$$

where $d_{i}(t)$ is a compact pulse train with energy concentrated near the origin, which describes the individual filtering effect of the *i*-th path.

The equivalent CIR of the final required solution is defined as

(17) $$h_{eff}\left(t\right)\;\equiv \;\sum_{i=1}^{P}a_{i}\cdot d_{i}(t-\tau_{i}),$$

$h_{eff}$ contains several sparse path clusters as well as tiny peak clusters. The internal structure of this peak cluster encodes the dispersion characteristics of each path. Despite this randomness, the overall form of the impulse-response model remains unchanged.

Substitute Equation (17) into Equation (15), and rewrite the received signal y as

(18) $$y\left(t\right)=s\left(t\right)*\left(\sum_{i=1}^{P}a_{i}\cdot d_{i}\left(t-\tau_{i}\right)\right)+n(t),$$

Through the above derivation, we have successfully converted a complex physical process with time-varying source signals mathematically into an equivalent standard LTI convolution model.

## 3. Sparse Blind Deconvolution Algorithm

### 3.1. Principle of the Algorithm

The well-posedness of blind deconvolution problem relies on effective prior constraints imposed by the physical process. In our application scenario, two key priors hold: (1) the CIR h is sparse due to discrete ground scattering, and (2) within the short duration of a single detection, the system can be accurately modeled as a LTI system. Based on these premises, the blind deconvolution problem is often transformed into solving a non-convex optimization problem with regularization constraints.

(19) $$\left(\hat{s},\hat{h}\right)=\arg\underset{s,h}{min}\left\{\frac{1}{2}{\left\|y-s*h\right\|}_{2}^{2}+\mathrm{Reg}(s,h)\right\},$$

where Reg(s,h) represents the regularization terms imposing prior knowledge on the source signal s and the CIR h.

To address the problem that traditional models cannot distinguish between quasi-periodic multipath structures and random scattering components, this paper proposes a three-variable model based on the idea of robust decomposition. This model no longer simply regards y as the sum of s∗h and white noise, but explicitly decomposes it into three physically different parts:

(20) $$y=\;s*h_{sp}\;+\;y_{cl}\;+\;n,$$

where $s*h_{sp}$ represents the main echo generated by the quasi-periodic multipath component with quasi-periodic structure, while $y_{cl}$ is specifically used to model the random scattering component, which contains random fluctuations and discrete clutter that deviates from the average behavior. L1 norm regularization is applied to $y_{cl}$ to constrain its energy and encourage its sparsity, thus separating it from the more energetic periodic echo.

Finally, we upgrade the optimization objective function of blind deconvolution from the traditional two-variable form to the following more complete and robust three-variable form, which forms the theoretical basis of the algorithm design:

(21) $$\underset{s,h_{sp},y_{cl}}{min}\;\left\{\frac{1}{2}{\left\|y-\;s*h_{sp}\;-\;y_{cl}\right\|}_{2}^{2}\;+\;R_{s}\left(s\right)\;+\;R_{h}\left(h_{sp}\right)\;+\;\lambda_{c}{\left\|y_{cl}\right\|}_{1}\right\},$$

where $R_{s}$ and $R_{h}$ are the priors of the source signal and sparse channel, respectively, and $\lambda_{c}\;>\;0$ is a regularization parameter that controls the sparsity of the estimated clutter component $y_{cl}$. The three-variable decomposition model forms the theoretical basis for all subsequent algorithm designs.

**Figure 3:**
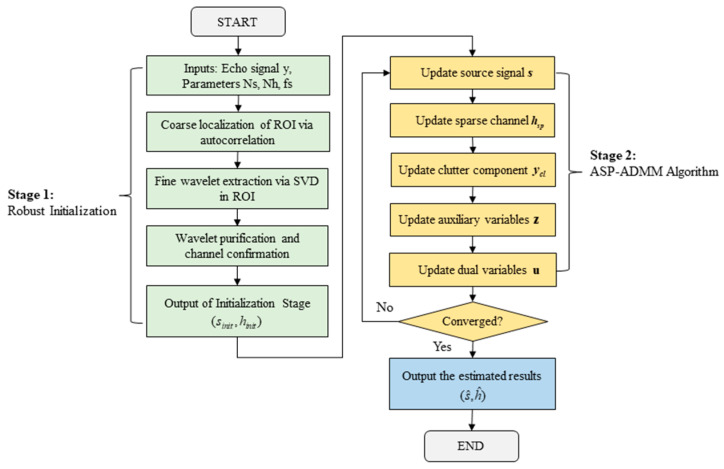
Algorithmic flowchart of the proposed SBD method.

Figure 3 illustrates the flowchart of our proposed method. The algorithm begins with a robust, data-driven initialization using autocorrelation and SVD to estimate a starting point. This estimate is then refined by our core ASP-ADMM, which iteratively solves for the source, channel, and clutter components.

### 3.2. Initialization Scheme

The performance of blind deconvolution algorithms is highly sensitive to initial values. Distorted initial values often lead to convergence to local optima. To address this, this paper adopts an SVD-based initialization strategy. First, the normalized autocorrelation function of the observed signal is computed, and a Savitzky–Golay smoothing filter is applied to suppress spurious peaks caused by noise. The position of the maximum peak in the smoothed autocorrelation function is then identified, serving as a preliminary estimate of the main echo energy center. A region of interest (ROI) for subsequent analysis is defined as a window centered at this maximum peak position, with a width *N* times the length of the source signal.

Within this ROI, the waveform of the source signal manifests as a recurring, dominant structural pattern. To facilitate data dimensionality reduction, the signal inside the ROI is segmented and arranged into a Hankel or Toeplitz matrix, where each column represents a signal frame of length $N_{s}$. SVD is then employed on this matrix to extract the principal components, effectively capturing the essential features of the source signal and providing a robust initial estimate for the blind deconvolution process.

After obtaining a high quality $s_{init}$, the channel estimate matching it is back-solved from the observed signal by Wiener filtered deconvolution.

(22) $$h_{\mathrm{init}}=\;F^{-1}\left\{\frac{F{\left\{s_{init}\right\}}^{*}\cdot F\left\{y\right\}}{{\left|F\left\{s_{init}\right\}\right|}^{2}+\lambda_{reg}}\right\},$$

where $F\left\{\cdot \right\}$ denotes the Fourier Transform operator, $F^{-1}$ its inverse, ${\left(\cdot \right)}^{*}$ represents the complex conjugate, and $\lambda_{reg}$ is a small regularization parameter to ensure numerical stability.

Finally, the obtained $h_{init}$ is subjected to a preliminary hard thresholding process to remove small noise components and obtain an initial estimate of a sparse channel. The Robust Initialization algorithm is shown in Algorithm 1.
**Algorithm 1** Robust Initialization Strategy1: **procedure** INITIALIZE_ROBUST
$(y,N_{s},N_{h},f_{s}$)
    //——Step 1: Coarse localization of ROI via Autocorrelation——2:
$\;\;\;\;\;\;\;\;\;\;\;c\left(\tau \right)\leftarrow XCORR(y,y)$.
3:
$\;\;\;\;\;\;\;\;\;\;\;\tau_{max}\leftarrow arg\;{max}_{\tau }\left|Smooth\left(c\left(\tau \right)\right)\right|.$ 4:
           Define Region of Interest (ROI) in y$\;\mathrm{centered}\;\mathrm{at}\;\tau_{max}$. 5:
    //——Step 2: Fine wavelet extraction via SVD in ROI—— 6:         Construct Hankel matrix X$\;\mathrm{from}\;\mathrm{frames}\;\mathrm{of}\;y_{ROI}$. 7: $\;\;\;\;\;\;\;\;\left[U,\Sigma ,V\right]\leftarrow SVD\left(X\right).$ 8: $\;\;\;\;\;\;\;\;s_{raw}\leftarrow U\left(:,1\right).$ % First left singular vector 9:
    //——Step 3: Wavelet purification and channel confirmation—— 10: $\;\;\;\;\;\;\;\;s_{init}\leftarrow P_{G}(s_{raw})$. % Project raw wavelet onto Gabor manifold 11: $\;\;\;\;\;\;\;\;H_{\mathrm{init}}(f)\;\leftarrow \;\frac{F{\left\{s_{init}\right\}}^{*}\cdot F\left\{y\right\}}{{\left|F\left\{s_{init}\right\}\right|}^{2}+\lambda_{reg}}.$ % Wiener deconvolution 12: $\;\;\;\;\;\;\;\;h_{\mathrm{init}}\;\leftarrow \;real\left(F^{-1}\left\{H_{init}(f)\right\}\right)$. 13: $\;\;\;\;\;\;\;\;h_{\mathrm{init}}\;\leftarrow \;HardThreshold\left(h_{init}\right).$ % Initial sparsification 14: 15: $\;\;\;\;\;\;\;\;\mathrm{return}\;s_{init},$$h_{init}$. 16: **end procedure**

### 3.3. ADMM Algorithm

The ADMM simplifies complex original problems by introducing auxiliary variables, breaking them down into a series of more manageable subproblems that are easier to solve. Specifically, it transforms the optimization objective into an equality-constrained problem by introducing auxiliary variables $z_{h}$ and $z_{c}$, along with imposing constraints $z_{h}\;=\;h_{sp}$ and $z_{c}\;=\;y_{cl}$. ADMM then solves this problem by minimizing its augmented Lagrangian function $L_{\rho }$.

(23) $$\begin{array}{cc} L_{\rho }\left(\left\{\cdot \right\}\right) & =\frac{1}{2}{\left\|y-s*h_{sp}-y_{cl}\right\|}_{2}^{2}+R_{s}\left(s\right)+R_{h}\left(z_{h}\right)+\lambda_{c}{\left\|z_{c}\right\|}_{1}\\ \; & +\langle u_{h},h_{sp}-z_{h}\rangle +\frac{\rho_{h}}{2}{\left\|h_{sp}-z_{h}\right\|}_{2}^{2}\\ \; & +\left\langle u_{c},y_{cl}-z_{c}\right\rangle +\frac{\rho_{c}}{2}{\left\|y_{cl}-z_{c}\right\|}_{2}^{2}\\ \; & \;s.t.{}^{\circ}\;\;s\in G, \end{array}$$

where {⋅} represents the set of all optimized variables, $u_{h}$ and $u_{c}$ are the Lagrangian dual variables of Scale-Form, and $\rho_{h}$ and $\rho_{c}$ are the corresponding quadratic penalty parameters.

In each iteration, s, $h_{sp}$, $y_{cl}$ and auxiliary variables z and u are updated alternately, the specific steps are as follows:

#### 3.3.1. Updating the Source Signal s

First, with $h^{k}$ fixed, solve for an s that primarily considers the data fidelity term. To enhance numerical stability and ensure the well-posedness of the subproblem, a minor regularization term is introduced. This term constrains the solution to remain within a neighborhood of the result from the previous iteration. The objective function for this step is:

(24) $$s^{k+1}\;=\;\arg\underset{s}{min}\frac{1}{2}{\left\|\left(y-y_{cl}^{k}\right)\;-\;s*h_{sp}^{k}\right\|}_{2}^{2}+\frac{\rho_{s}}{2}{\left\|s-\;s^{k}\right\|}_{2}^{2},$$

where $\rho_{s}$ is a small positive constant. Equation (24) is a standard quadratic programming problem, and its solution satisfies the following normal equation.

(25) $$\left({\left(H_{sp}^{k}\right)}^{T}H_{\mathrm{sp}}^{k}+\rho_{s}I\right)s={\left(H_{sp}^{k}\right)}^{T}\left(y-y_{cl}^{k}\right)+\rho_{s}s^{k},$$

where $H_{sp}^{k}$ is the convolution matrix constructed from $h_{sp}^{k}$. Considering the large dimension of the linear system, the preconditioned conjugate gradient method (PCG) is used for efficient iterative solution, where the calculation of the matrix-vector product ${\left(H_{sp}^{k}\right)}^{T}H_{sp}^{k}s$ can be accelerated by fast Fourier transform (FFT).

Next, project $s^{k+1}$ onto the physically feasible set G defined by the Gabor atom model g(θ). This process can be achieved by solving a nonlinear least-squares fitting problem.

(26) $$\theta^{k+1}=\arg\underset{\theta }{min}{\left\|s^{k+1}-g(\theta )\right\|}_{2}^{2},$$

where θ is the physical parameter of the Gabor model. Equation (26) is efficiently solved using the Levenberg–Marquardt algorithm with bound constraints, and the solution is updated to the normalized variable $s^{k+1}\;=\;g(\theta^{k+1})/{\left\|g(\theta^{k+1})\right\|}_{2}$. This projection step provides a clean and noise-free known quantity for the subsequent update of h.

#### 3.3.2. Updating the Channel Impulse Response $h_{sp}$

With $s^{k+1}$ fixed, the update of $h_{sp}$ is expressed as an augmented Lagrange function, in the form as follows.

(27) $$h_{sp}^{k}=\arg\underset{h_{sp}}{min}\frac{1}{2}{\left\|\left(y-y_{cl}^{k}\right)-s^{k+1}*h_{sp}\right\|}_{2}^{2}+\frac{\rho_{h}}{2}{\left\|h_{sp}-\left(z_{h}^{k}-u_{h}^{k}\right)\right\|}_{2}^{2},$$

Similarly, Equation (27) constitutes a QP problem, which is efficiently solved using the PCG algorithm.

#### 3.3.3. Updating the Clutter Component $y_{cl}$

The update of the clutter component $y_{cl}$ also constitutes a separable QP problem, whose gradient-zero condition leads to the following closed-form solution:

(28) $$y_{cl}^{k+1}\;=\;\frac{1}{1+\rho_{c}}\left(\left(y-\;s^{k+1}*h_{sp}^{k+1}\right)\;+\;\rho_{c}\left(z_{c}^{k}\;-\;u_{c}^{k}\right)\right),$$

#### 3.3.4. Updating the Auxiliary Variable z

Apply the Laplacian Sparse prior (LSP) to variable h, which is efficiently implemented via its proximal operator possessing a closed-form solution.

(29) $$z_{h}^{k+1}=\arg\underset{z_{h}}{min}\lambda_{h}R_{h}\left(z_{h}\right)+\frac{\rho_{h}}{2}{\left\|z_{h}-(h_{sp}^{k+1}+u_{h}^{k})\right\|}_{2}^{2},$$

The update of $z_{h}$ represents the core procedure for imposing the quasi-periodic structure prior of the channel. To achieve this, we develop a two-step solver. The first step is structure determination: based on the period T estimated from spectral analysis of the observed signal y, the solver identifies an optimal periodic support set S in the current iterative signal $v_{h}\;=\;h_{sp}^{k+1}\;+\;u_{h}^{k}$ by applying a maximum energy criterion. This support set consists of a series of neighborhood windows of width W. The window width W is automatically set according to the full width at half maximum of the autocorrelation function of the source signal, obtained during the initialization phase, so as to match the effective duration of the source signal. The second step is amplitude optimization: after determining the hard support set S, the algorithm applies the LSP proximal operator exclusively to the corresponding components of $v_{h}$ within S to promote sparsity, while all coefficients outside the support set are forced to zero.

(30) $$z_{h}^{k+1}=P_{T,W}^{\mathrm{LSP}}\left(v_{h}\right)\;\equiv \;M_{S}⊙{\mathrm{prox}}_{\lambda_{h}/\rho_{h}}^{\mathrm{LSP}}\left(M_{S}⊙v_{h}\right),$$

where $M_{S}$ is a binary mask defined by the support set S, where its entries are 1 within Sand 0 elsewhere. The symbol ⊙ denotes the Hadamard (element-wise) product. This operation first uses the mask to extract the components of $v_{h}$ inside the support set, applies the LSP proximal operator to them, and then places the results back to their original positions via the same mask, thereby enforcing the hard constraint of setting coefficients outside S to zero.

For the clutter component $y_{cl}$, the algorithm employs a standard L1-norm sparsity prior to constrain its energy and promote sparsity. The subproblem for updating its auxiliary variable $z_{c}$ is

(31) $$z_{c}^{k+1}=\arg\underset{z_{c}}{min}\lambda_{c}{\left\|z_{c}\right\|}_{1}+\frac{\rho_{c}}{2}{\left\|z_{c}-(y_{cl}^{k+1}+u_{c}^{k})\right\|}_{2}^{2},$$

The solution to this problem is the classic soft thresholding operator.

(32) $$z_{c}^{k+1}=S_{\lambda_{c}/\rho_{c}}\left(y_{cl}^{k+1}+u_{c}^{k}\right),$$

where $S_{\tau }(x{)}_{i}\;=\;\mathrm{sign}\left(x_{i}\right)\max(\left|x_{i}\right|\;-\;\tau ,0)$.

#### 3.3.5. Updating the Dual Variable u

Update the dual variables to drive the consensus between the local variables $h_{sp}$, $y_{cl}$ and the global auxiliary variables z.

(33) $$u_{h}^{k+1}=u_{h}^{k}+h_{sp}^{k+1}-z_{h}^{k+1},\;u_{c}^{k+1}=u_{c}^{k}+y_{cl}^{k+1}-z_{c}^{k+1},$$

By alternately executing the above steps, the algorithm will gradually converge to a stable solution that satisfies all constraints and priors. The pseudo-code of the ASP-ADMM algorithm is presented in Algorithm 2.
**Algorithm 2** Proposed Asymmetric Structured Prior ADMM Algorithm1: **procedure**
$\mathrm{ASP\_ADMM}(y,N_{s},N_{h},f_{s},\;parameters$)
    //——Phase 1: Initialization——2:
$\;\;\;\;\;\;\;\;\;\;\;\;\left[s^{0},h_{sp}^{0}\right]\leftarrow INITIALIZE\_ROBUST(y,N_{s},N_{h},f_{s})$.
3: $\;\;\;\;\;\;\;\;\;\;\;\;\mathrm{Initialize}\;y_{cl}^{0},\;z_{h}^{0},z_{c}^{0},u_{h}^{0},u_{c}^{0}\;\mathrm{based}\;\mathrm{on}\;\mathrm{initial}\;\mathrm{values}.$
    //——Phase 2: ADMM Iteration—— 4:             for k=0$\;\mathrm{to}\;K_{max}-1$
**do** 5:                    //——Step 2.1: Update Source Signal s 6: $\;\;\;\;\;\;\;\;\;\;\;\;\;\;\;\;\;\;\;s_{updated}\;\leftarrow \;\arg\underset{s}{min}\frac{1}{2}{\left\|\left(y-y_{cl}^{k}\right)\;-\;s*h_{sp}^{k}\right\|}_{2}^{2}+\frac{\rho_{s}}{2}{\left\|s-\;s^{k}\right\|}_{2}^{2}$. 7: $\;\;\;\;\;\;\;\;\;\;\;\;\;\;\;\;\;\;\;s^{k+1}\leftarrow P_{G}(s_{updated})$. 8: $\;\;\;\;\;\;\;\;\;\;\;\;\;\;\;\;\;\;\;//——\mathrm{Step}\;2.2:\;\mathrm{Update}\;\mathrm{Sparse}\;\mathrm{Channel}\;h_{sp}$ 9: $\;\;\;\;\;\;\;\;\;\;\;\;\;\;\;\;\;\;\;\;h_{sp}^{k}\;\leftarrow \;\arg\underset{h_{sp}}{min}\frac{1}{2}{\left\|\left(y-\;y_{cl}^{k}\right)\;-\;s^{k+1}*h_{sp}\right\|}_{2}^{2}\;+\;\frac{\rho_{h}}{2}{\left\|h_{sp}\;-\;\left(z_{h}^{k}\;-\;u_{h}^{k}\right)\right\|}_{2}^{2}$. 10: $\;\;\;\;\;\;\;\;\;\;\;\;\;\;\;\;\;//——\mathrm{Step}\;2.3:\;\mathrm{Update}\;\mathrm{Clutter}\;\mathrm{Component}\;y_{cl}$ 11: $\;\;\;\;\;\;\;\;\;\;\;\;\;\;\;\;\;\;y_{cl}^{k+1}\;\leftarrow \;\frac{1}{1+\rho_{c}}\left(\left(y-\;s^{k+1}*h_{sp}^{k+1}\right)\;+\;\rho_{c}\left(z_{c}^{k}\;-\;u_{c}^{k}\right)\right)$ 12:                  //——Step 2.4: Update Auxiliary Variables z 13: $\;\;\;\;\;\;\;\;\;\;\;\;\;\;\;\;\;\;v_{h}\;\leftarrow \;h_{sp}^{k+1}\;+\;u_{h}^{k}$. 14: $\;\;\;\;\;\;\;\;\;\;\;\;\;\;\;\;\;\;z_{h}^{k+1}\;\leftarrow \;P_{T,W}^{\mathrm{LSP}}\left(v_{h}\right)$. 15: $\;\;\;\;\;\;\;\;\;\;\;\;\;\;\;\;\;\;z_{c}^{k+1}\;\leftarrow \;S_{\lambda_{c}/\rho_{c}}\left(y_{cl}^{k+1}\;+\;u_{c}^{k}\right)$. 16:                  //——Step 2.5: Update Dual Variables u
17:
$\;\;\;\;\;\;\;\;\;\;\;\;\;\;\;\;\;\;u_{h}^{k+1}\;\leftarrow \;u_{h}^{k}+h_{sp}^{k+1}-z_{h}^{k+1}$
18:
$\;\;\;\;\;\;\;\;\;\;\;\;\;\;\;\;\;\;u_{c}^{k+1}\;\leftarrow \;u_{c}^{k}+y_{cl}^{k+1}-z_{c}^{k+1}$
19:                 //——Step 2.6: Check for Convergence
20: $\;\;\;\;\;\;\;\;\;\;\;\;\;\;\;\;\;\;\epsilon_{s}^{k+1}\;\leftarrow \;{\left\|s^{k+1}\;-\;s^{k}\right\|}_{2}/{\left\|s^{k}\right\|}_{2}$.21: $\;\;\;\;\;\;\;\;\;\;\;\;\;\;\;\;\;\;\epsilon_{h}^{k+1}\;\leftarrow \;{\left\|h_{sp}^{k+1}\;-\;h_{sp}^{k}\right\|}_{2}/{\left\|h_{sp}^{k}\right\|}_{2}$.22:
$\;\;\;\;\;\;\;\;\;\;\;\;\;\;\;\;\;\;\mathrm{if}\;\epsilon_{s}^{k+1}<\tau_{tol}$
$\;\mathrm{and}\;\epsilon_{h}^{k+1}<\tau_{tol}$ 
**then**
23:                         break24:                   end if25:                end for26:
$\;\;\;\;\;\;\;\;\;\;\;\;\;\;\;\mathrm{return}\;s^{k+1},\;h_{sp}^{k+1},y_{cl}^{k+1}$.27: **end procedure**

### 3.4. Convergence and Performance Analysis

In this section, we provide a theoretical analysis of the proposed ASP-ADMM algorithm’s key properties. We first discuss its convergence behavior and the basis for its recovery guarantees and then analyze its computational complexity.

#### 3.4.1. Convergence and Recovery Guarantees

The convergence of the proposed ASP-ADMM algorithm is analyzed within the framework of multi-block alternating minimization for non-convex objectives. Since the problem involves non-convex terms (e.g., the Gabor manifold constraint on the source signal and the periodic sparse prior on the channel), standard convex ADMM convergence guarantees do not apply. Our analysis establishes convergence by verifying three key properties [32]: (i) sufficient decrease in the augmented Lagrangian, (ii) convergence of successive iterates, and (iii) satisfaction of the optimality conditions at limit points.

Let $L_{\rho }$ denote the augmented Lagrangian defined in Equation (22), and let $Z^{\left(k\right)}$ represent the set of all primal and dual variables at iteration k.

**Lemma 1 (Sufficient Decrease).** 
*Suppose the penalty parameters $\rho_{s}$, $\rho_{h}$, $\rho_{c}$ are sufficiently large. Then, the sequence $\left\{L_{\rho }\left(Z^{\left(k\right)}\right)\right\}$ is monotonically non-increasing. Moreover, there exists a constant C>0 such that for all k:*

(34) $$L_{\rho }\left(Z^{\left(k\right)}\right)-L_{\rho }\left(Z^{\left(k+1\right)}\right)\;\geq \;C\left({\left\|s^{\left(k+1\right)}-s^{\left(k\right)}\right\|}_{2}^{2}+{\left\|{h_{sp}}^{\left(k+1\right)}-{h_{sp}}^{\left(k\right)}\right\|}_{2}^{2}+{\left\|{y_{cl}}^{\left(k+1\right)}-{y_{cl}}^{\left(k\right)}\right\|}_{2}^{2}\right)\;,$$

*The descent in $L_{\rho }$ arises from the block updates. The subproblems for $h_{sp}$ and $y_{cl}$ are strongly convex, ensuring a decrease proportional to the squared step size. For the non-convex s-update, the proximal term $\frac{\rho_{s}}{2}{\left\|s-s^{k}\right\|}_{2}^{2}$ guarantees sufficient descent when $\rho_{s}$ exceeds the Lipschitz constant of the gradient of the data fidelity term with respect to s. A similar argument applies to the update of $z_{h}$ via the periodic sparse projection. Summing the descent from all blocks yields the result.*

**Proposition 1 (Convergence to a Critical Point).** 
*Assume the objective function is bounded below and the sequence $\left\{Z^{\left(k\right)}\right\}$ is bounded. Then, the sequence generated by ASP-ADMM converges to a critical point of the objective function defined in Equation (21).*

*From Lemma 1 and the boundedness below of $L_{\rho }$, the sequence $\left\{L_{\rho }\left(Z^{\left(k\right)}\right)\right\}$ converges. Lemma 1 then implies:*

(35) $${lim}_{k\to \infty }\left({\left\|s^{\left(k+1\right)}-s^{\left(k\right)}\right\|}_{2}^{2}+{\left\|{h_{sp}}^{\left(k+1\right)}-{h_{sp}}^{\left(k\right)}\right\|}_{2}^{2}+{\left\|{y_{cl}}^{\left(k+1\right)}-{y_{cl}}^{\left(k\right)}\right\|}_{2}^{2}\right)=0\;,$$

*Combined with the dual update rule in Equation (33), this leads to the convergence of primal residuals:*

(36) $${lim}_{k\to \infty }{\left\|{h_{sp}}^{\left(k\right)}\;-\;{z_{h}}^{\left(k\right)}\right\|}_{2}=0,\;\;\;{lim}_{k\to \infty }{\left\|{y_{cl}}^{\left(k\right)}-{z_{c}}^{\left(k\right)}\right\|}_{2}=0\;,$$

Let $Z^{*}$ be a limit point of $\left\{Z^{\left(k\right)}\right\}$. The conditions in (35) and (36), together with the first-order optimality condition of each subproblem, ensure that $Z^{*}$ satisfies the KKT conditions of the original problem, and is therefore a critical point.

While convergence to a single point can be strengthened via the Kurdyka-Łojasiewicz (KL) property, the above establishes essential convergence to a meaningful solution. The practical effectiveness of the solution is further supported by our robust initialization and physically grounded priors.

#### 3.4.2. Computational Complexity Analysis

The computational cost of the proposed ASP-ADMM algorithm is primarily determined by the iterative updates within its main loop, where the bottleneck lies in the subproblems for updating the source signal s and the sparse channel $h_{sp}$. Each of these updates requires solving a large-scale linear system (e.g., Equations (24) and (27)), which would be computationally prohibitive if implemented naively via explicit matrix operations. To ensure efficiency, we employ a PCG solver, where all matrix-vector products involving the underlying Toeplitz convolution matrices are computed in the frequency domain using FFT. This approach yields a complexity of $O(K_{pcg}N_{conv}logN_{conv})$ for each subproblem, where $N_{conv}=N_{s}+N_{h}-1$ is the length of the linear convolution and $K_{pcg}$ is the number of inner PCG iterations.

Consequently, the dominant cost per outer ADMM iteration is the sum of these two subproblems. The remaining update steps, such as the closed-form solution for the clutter term $y_{cl}$ and the element-wise operations for auxiliary and dual variables, exhibit a linear complexity of $O(N_{conv})$, which is asymptotically subdominant. Thus, the total complexity is $O({K_{admm}\cdot K}_{pcg}\cdot N_{conv}logN_{conv})$, where $K_{admm}$ is the number of outer ADMM iterations required for convergence. Although the specific values of $K_{admm}$ and $K_{pcg}$ depend on problem conditioning, the crucial takeaway is the algorithm’s quasi-linear scaling with respect to the signal dimension, governed by the $O(N_{conv}logN_{conv})$ term.

This complexity order is fundamentally tied to the use of FFT for convolution, which is the same core operation that dictates the cost of standard fast non-blind deconvolution algorithms (e.g., ISTA, FISTA). Consequently, the proposed framework’s computational cost is on par with these standard methods, demonstrating that despite the added challenge of blindness, the algorithm remains computationally efficient and scalable.

## 4. Simulation Results

### 4.1. Simulation Data Experiment and Analysis

To quantitatively characterize the severity of temporal overlap among multipath components and thereby depict the ill-posedness of the inverse problem, this paper introduces a resolution factor. This factor compares the minimum path delay separation Δτ in the CIR with the system’s inherent delay resolution 1/BW, defining it explicitly as their ratio:

(37) $$\kappa_{res}\;=\;\frac{\Delta \tau_{min}}{\tau_{res}}\;=\;\Delta \tau_{min}\cdot BW,$$

According to Equation (11), it can be concluded that $\Delta \tau_{min}=1/f_{c}$, indicating that the resolution factor is approximately equal to the relative bandwidth of the source signal.

(38) $$\kappa_{res}=\Delta \tau_{min}\cdot BW=\frac{f_{H}-f_{L}}{f_{c}},$$

where $f_{H}$ and $f_{L}$ denote the −10 dB cutoff frequencies.

When $\kappa_{res}\;>\;1$, all multipath components are resolvable in principle, and the corresponding inverse problem is relatively well-conditioned.

When $\kappa_{res}\;\leq \;1$, the time delay between at least two paths is less than or equal to the system’s Rayleigh resolution limit. In this scenario, classical linear methods will fail, and the problem enters the super-resolution region. A smaller value of $\kappa_{res}$ indicates a more ill-conditioned problem, placing higher demands on the recovery algorithm’s ability to leverage prior knowledge.

Since the real source signal s and channel response h are unknown, to verify and evaluate the performance of the algorithm proposed in this paper, simulation data is constructed based on the following LTI model.

(39) $$y=s_{true}*h_{true}+n,$$

The source signal s is represented by Gabor pulses, making it easy to control different center frequencies and pulse widths. Its discrete time is defined as

(40) $$s_{true}\left[n\right]=\exp\left(-\frac{{\left(n\Delta t\right)}^{2}}{2\sigma_{s}^{2}}\right)\cos\left(2\pi f_{c}n\Delta t+\phi_{s}\right),n\in \left[-N_{s}/2,\ldots ,N_{s}/2-1\right],$$

where $\Delta t\;=\;1/f_{s}$ is the sampling period and $N_{s}$ is the effective length of the signal.

The channel response h is modeled as an ideal, equally spaced sparse pulse train.

(41) $$h_{true}\left[n\right]=\sum_{k=0}^{K_{p}-1}a_{k}\cdot \delta \left[n-\left(p_{0}+kT\right)\right],$$

where $p_{0}$ is the initial delay of the first path, T is the fixed delay interval between paths, and $K_{p}$ is the number of sparse paths.

The amplitude $a_{k}$ of the *k*-th pulse is jointly modulated by macroscopic attenuation and microscopic randomness. It can be obtained by multiplying a deterministic exponential attenuation envelope with a random lognormal fluctuation. The attenuation rate is controlled by the time constant $\tau_{decay}$, and the intensity of the lognormal fluctuation is controlled by $\sigma_{ln}$.

(42) $$a_{k}=\mathrm{sign}\left(N\left(0,1\right)\right)\cdot \exp\left(-\frac{\left(p_{0}+kT\right)\Delta t}{\tau_{decay}}\right)\cdot 10^{\frac{N\left(0,\sigma_{ln}^{2}\right)}{20}},$$

To systematically evaluate the algorithm’s performance across varying resolution factors, this study maintains the quasi-periodic structure of the channel (i.e., fixes the minimum path separation $\Delta \tau_{min}$) and achieves continuous control over $\kappa_{res}$ by adjusting the effective bandwidth of the source signal s. Specifically, based on Equation (40), the time-domain pulse width of s is progressively broadened while holding its center frequency $f_{c}$ constant. Leveraging the time-bandwidth inverse proportionality principle of the Fourier transform, this operation generates a series of source waveforms with distinct system resolutions. Finally, convolving these resolution-varying source signals with a fixed CIR produces a comprehensive test dataset spanning from the resolvable region to the super-resolution region.

**Figure 4:**
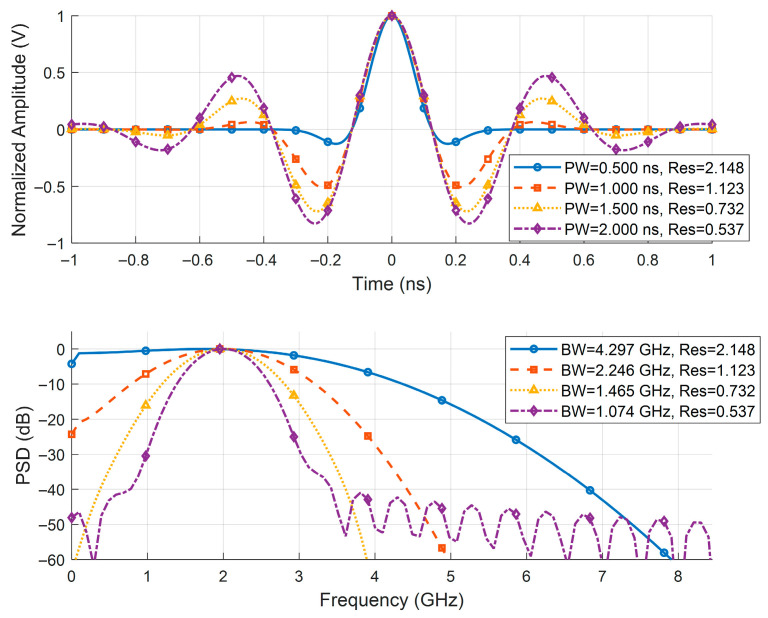
Source Signal Waveform and Power Spectral Density at Different System Resolutions.

To fairly compare the proposed algorithm with SOOT, MCMC, and L1-ADMM, all experiments share unified settings: the impulse response length is Nh = 500, containing 49 evenly spaced non-zero pulses with interval 10 and amplitudes given by Equation (42). Source signals of different bandwidths are used to represent various resolution conditions. The specific source signals are shown in Figure 4. Gaussian white noise is added with SNR levels of {5,10,15,20,25,30} dB. Each algorithm performs 50 Monte Carlo trials per SNR, with a maximum of 1000 iterations and convergence tolerance $10^{-3}$. We define a recovered sparse sequence $\hat{h}$ as a successful estimate if its Normalized Mean Square Error (NMSE) with respect to the ground truth h is less than a given threshold τ.

(43) $$NMSE\left(\hat{h},h\right)=\frac{{\left\|\hat{h}-h\right\|}^{2}}{{\left\|h\right\|}^{2}}\leq \tau ,$$

The prior configurations of the compared algorithms are summarized as follows:

- SOOT: assumes known impulse response length Nh and uses a smoothed ℓ1/ℓ2 sparsity regularizer, with parameters tuned via validation to minimize NMSE.
- MCMC: assumes Nh and an upper bound on sparsity, adopting the spike-and-slab prior with parameters validated for optimal recovery.
- L1-ADMM: the same initialization as the proposed algorithm, but with the periodic projection prior on h replaced by an ℓ1 sparsity regularizer.

All algorithms are evaluated under identical noise realizations and random seeds for paired comparisons. For the baseline methods, all respective hyperparameters were individually optimized via validation to ensure a fair comparison, whereas for our proposed method, they were fixed to $\lambda_{h}\;=\;2\;\times \;10^{-4},{\;\rho }_{h}\;=\;1.0$ and ${\;\rho }_{c}\;=\;1.0$ for simulation scenarios. To remove the influence of amplitude scaling and time shift, all estimates are aligned before computing NMSE. A trial is considered successful if NMSE ≤ 0.2, and the success rate is reported for each SNR. Experiments are repeated with different source bandwidths to assess robustness and recoverability under super-resolution conditions.

**Figure 5:**
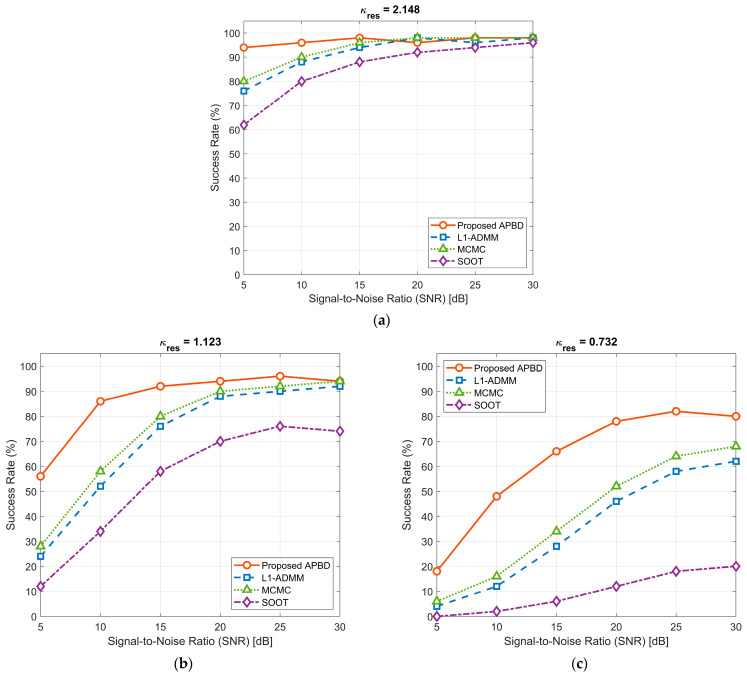
Success rate of different blind deconvolution algorithms versus SNR under three distinct resolution factor: (**a**) $\kappa_{res}\;=\;2.148$; (**b**) $\kappa_{res}\;=\;1.123$; (**c**) $\kappa_{res}\;=\;0.732$.

The experimental results, shown in Figure 5, clearly demonstrate that the alignment between the prior model and the problem structure has a decisive impact on recovery performance. In the resolvable region with $\kappa_{res}\;>\;1$, all sparsity-based methods perform well. However, $\kappa_{res}$ drops below 1, the ill-posedness of the problem intensifies, and performance differences become pronounced. L1-ADMM and MCMC, which rely on generic random sparsity priors, struggle to stably separate closely spaced paths under highly correlated sensing matrices, leading to a rapid decline in success rates. SOOT performs the worst due to fundamental mismatches between its physical model and the temporal structure of the channel. In contrast, the proposed algorithm employs a periodic-structure projection operator to precisely constrain the solution space onto the correct low-dimensional manifold. Even in the deep super-resolution scenario of $\kappa_{res}\;=\;0.732$, the success rate remains stably above 80% for SNRs exceeding 20 dB, demonstrating outstanding robustness. These results further confirm that directly embedding a strong, structure-specific prior into the optimization process is the most effective approach for addressing such extreme recovery problems.

### 4.2. Measured Data Experiment and Analysis

**Figure 6:**
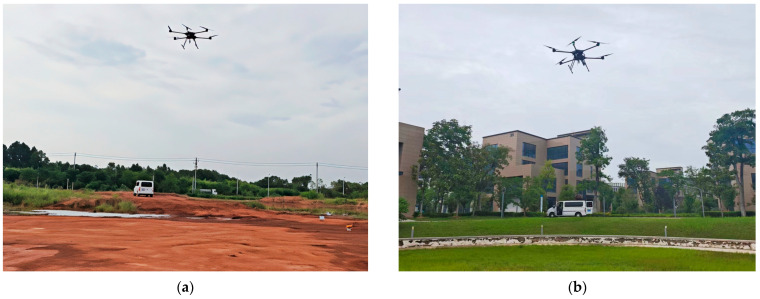
Field data acquisition scenarios. (**a**) smooth soil ground; (**b**) grassland.

For validation on measured data, a comprehensive dataset was acquired using a UWB fuze system. The system’s receiver architecture is based on ETS, which leverages Doppler frequency shift for high-resolution waveform reconstruction. To generate the required relative motion during experiments, the fuze system was mounted on an unmanned aerial vehicle (UAV) that executed vertical descent maneuvers over the ground targets, simulating terminal engagement scenarios. A total of 20 independent measurements were acquired for each of two distinct ground surfaces: a smooth soil ground referred to as Surface A, and a grassland referred to as Surface B. The final segment consisting of 870 samples, recorded at a sampling frequency of 2.5 kHz, was retained from each measurement for subsequent analysis (see Figure 6).

Before performing blind deconvolution on the truly measured ground echo signals, it is necessary to analyze the internal structure of the signals first. In this paper, cepsogram analysis is adopted to transform the convolution relationship in the time domain into a linear additive relationship in the cepsogram domain, thereby effectively separating the contributions of the source signal and the channel.

**Figure 7:**
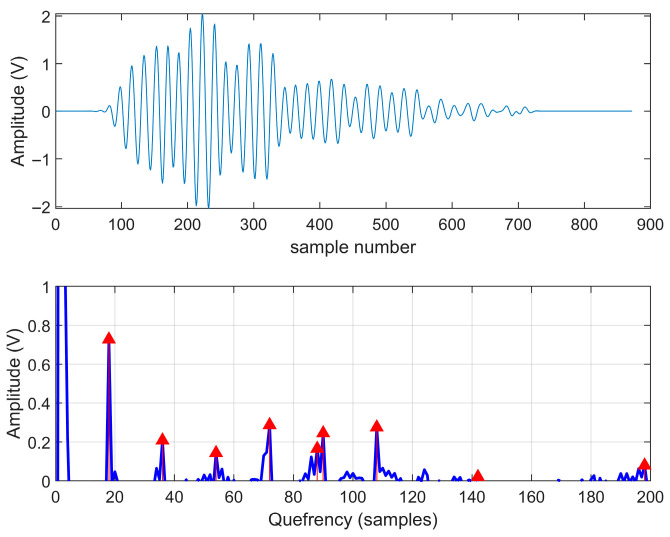
The measured waveform and cepstrum of the observed signal.

It can be observed that in the low-quefrency region of the cepstrum, there exists an isolated dominant peak with highly concentrated energy, a feature consistent with the cepstral morphology of short-duration, aperiodic pulses. In the high-quefrency region, the cepstrum exhibits a series of distinct and approximately equally spaced harmonic peaks, as marked by the red triangles in Figure 7. Since the contribution of the short-duration pulse in this region has largely decayed to zero, these periodic peaks must originate from the CIR. Furthermore, the decaying trend of these peaks corroborates that the channel exhibits physically plausible energy attenuation characteristics. Therefore, cepstrum analysis empirically demonstrates that the observed signal results from the convolution of a short-duration pulse component and a channel component with inherent periodic structure. This finding provides direct and compelling justification for subsequent algorithms to incorporate periodic structural priors in modeling the channel.

The method proposed in this paper is used to perform blind deconvolution on the observed signal to obtain the source signal s and the CIR h.

**Figure 8:**
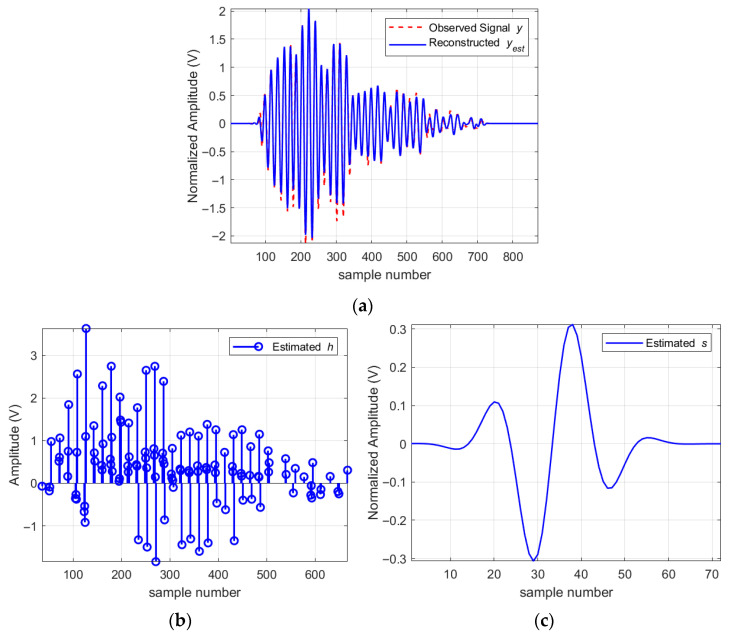
Blind deconvolution results for observed signal. (**a**) The reconstructed signal (blue solid line) demonstrates an excellent fit to the original observed signal y (red dashed line); (**b**) The recovered CIR h; (**c**) The recovered source signal s.

As can be seen from Figure 8, the amplitude envelope of the CIR is basically consistent with the envelope of the observed signal. Due to the parametric prior, the curve of the source signal is very smooth. The resolution factor calculated according to Equation (38) is 0.751. Therefore, the CIR of the measured data is super-resolution, which gives full play to the advantages of the method proposed in this paper. This recovered CIR provides a clear manifest of the individual multipath components, transforming the ranging problem from peak detection on an ambiguous waveform to a more informed decision-making process based on identifying the true range-marker, such as the first significant path, within the now-decoupled channel structure.

To independently verify the effectiveness and consistency of the algorithm, the recovery results are analyzed in the frequency domain. The core lies in verifying whether the solution pairs $\left(\hat{s},\hat{h}\right)$ follow the convolution theorem $Y\left(f\right)\;\approx \;\hat{S}(f)\hat{H}(f)$. For this purpose, the channel transfer function $\hat{H}(f)$ directly restored by the algorithm is compared with the $Y(f)/\hat{S}(f)$ indirectly inferred.

**Figure 9:**
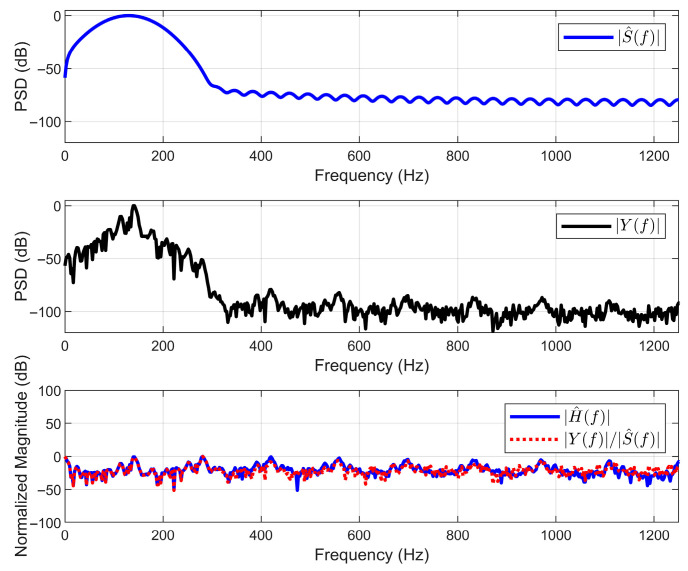
Frequency-domain validation of the blind deconvolution results.

As can be seen from Figure 9, the spectrum $|\hat{S}(f){|}^{2}$ of the restored source signal presents a physically reasonable smooth broadband function, while the spectrum $|Y(f){|}^{2}$ of the observed signal shows a quasi-periodic harmonic structure under its envelope. The directly restored $\hat{H}(f)$ and the indirectly inferred $Y(f)/\hat{S}(f)$ show a high consistency in the main harmonic structure, and the deviation part mainly stems from the influence of noise. This result proves that the algorithm has successfully decoupled a solution pair that is consistent with the convolution theorem. Furthermore, the approximate comb spectral form presented by the reduced $\hat{H}(f)$ not only explains the harmonic source of Y(f), but also, in turn, confirms the validity of the adopted quasi-periodic structure prior.

To evaluate the recovery consistency of this algorithm when processing different measured data, we performed blind deconvolution on the measured data of two types of ground echo signals.

**Figure 10:**
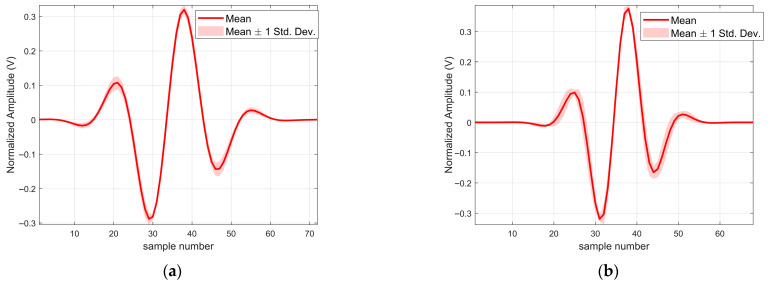
Recovered source signals from the measured data of two types of ground echo signals: (**a**) Surface A; (**b**) Surface B.

As shown in Figure 10, the source signals recovered from the measured data of the same ground type (represented by the translucent curves in Figure 10a,b) demonstrate a high degree of consistency, clustering closely around their respective clear average waveforms (solid red lines). Moreover, a comparison of the average waveforms for the two ground types reveals a strong similarity in both waveform structure and envelope morphology (see Table 1).

**Figure 11:**
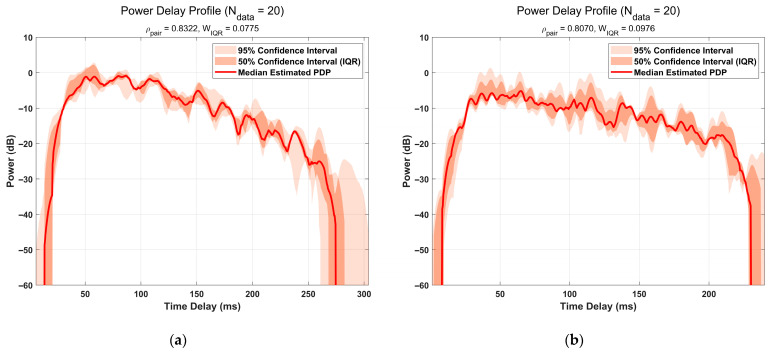
Smoothed Power Delay Profile (PDP) ensembles for measured data of two types of ground echo signals: (**a**) Surface A; (**b**) Surface B.

To quantitatively evaluate the consistency of the algorithm in processing multiple sets of real measurements, the average correlation coefficients of the source signals recovered from two sets of measured signals were first calculated, reaching 0.9938 and 0.9956, respectively. This indicates that the algorithm can robustly recover the inherent morphological structure of the source signals from different observations with high stability. Secondly, the relative standard deviation norms were computed as 0.1296 and 0.2099, respectively. This suggests that, despite the randomness and physical variations introduced by different measurements, the overall point-by-point fluctuation of the recovery results is effectively constrained within an extremely small range (see Figure 11).

The macroscopic structure of the recovered CIR was analyzed using its PDP, where the instantaneous power was smoothed with a Gaussian window for a more stable energy trend. Figure 10 shows a high visual consistency of the PDPs for both ground types. To quantify this consistency and the fine-waveform similarity of the CIRs, respectively, the relative IQR width of 20 PDP measurements and the average pairwise correlation coefficient of 20 CIR measurements were computed. The results are summarized in Table 2.

The quantitative results demonstrate the algorithm’s efficacy: the low relative IQR widths (0.0775 and 0.0976) indicate stable recovery of the macroscopic energy envelope. Furthermore, the high average pairwise correlation coefficients (0.8322 and 0.8070) validate its capability to accurately reconstruct the fine-grained waveform structure.

## 5. Conclusions

To address the severe ranging ambiguity in UWB fuzes caused by multipath delay spread, this paper proposes and validates a blind deconvolution framework based on an asymmetric structural prior. The core contribution of this method lies in providing a tool for channel structure extraction, where the recovered fine-grained multipath information offers essential theoretical insights and analytical foundations for fundamentally understanding and resolving the ranging ambiguity induced by signal aliasing. The simulation experiments verify the effectiveness and robustness of the framework, thereby providing the necessary confidence for its application to measured data. The main conclusions of the simulation and actual measurement analysis are as follows:

First, we established and validated an equivalent discrete multipath model to effectively characterize the ground CIR. We further confirm the hypothesized quasi-periodic structure of the channel through cepstral analysis of measured signals. The cepstrum of real echo data clearly shows distinct, evenly spaced harmonic peaks, providing direct and data-driven evidence of the channel’s inherent periodicity. This key finding provides both physical and empirical justifications for discarding generic random sparsity assumptions and designing a targeted periodic structural prior.

Second, in the challenging deep super-resolution scenarios ($\kappa_{res}\;<\;1$) encountered with the measured data, the proposed framework successfully recovered the underlying periodic channel structure, whereas conventional algorithms failed. This result demonstrates the decisive advantage of our novel periodic sparse cluster projection operator in overcoming fundamental resolution limits.

Finally, analysis of multiple measured datasets confirmed the algorithm’s high consistency and repeatability. The recovered source signal exhibited high morphological consistency. Concurrently, the channel PDPs showed excellent stability in their macroscopic energy structure. Together, these results validate the framework’s ability to robustly decouple a stable source signal from a consistent channel representation in real-world scenarios.

These findings highlight the algorithm’s strength in channel structure extraction, positioning it primarily as a powerful offline analysis tool. This perspective guides future work in two key directions: leveraging the recovered CIRs as a benchmark for developing improved real-time ranging algorithms and using their statistical properties for terrain characterization to enable future terrain-adaptive fuzes. While the current method is best suited for uniform terrains, extending it to non-uniform scatterers remains an important challenge. Ultimately, this work provides a foundational methodology for understanding and overcoming ranging ambiguity in UWB fuzes.

## Figures and Tables

**Table 1:** Quantitative Metrics for the Consistency of Recovered s from Measured Data.

Parameter	Measured Data1	Measured Data2
Mean Correlation Coeff	0.9938	0.9856
Relative Standard Deviation Norm	0.1296	0.2099

**Table 2:** Quantitative Metrics for the Consistency of Recovered h from Measured Data.

Parameter	Measured Data1	Measured Data2
Relative IQR Width	0.0775	0.0976
Average pairwise correlation coefficient	0.8322	0.8070

## Data Availability

The data are available from the corresponding author upon reasonable request.

## Abbreviations

The following abbreviations are used in this manuscript:

UWB	Ultra-wideband
SNR	Signal-to-Noise Ratio
ADMM	Alternating Direction Method of Multipliers
EDMM	Equivalent Discrete Multipath Model
SBD	Sparse Blind Deconvolution
TOF	Time of Flight
CIR	Channel Impulse Response
ETS	Equivalent Time Sampling
SOOT	Smooth One-Over-Two norm ratio
MCMC	Markov Chain Monte Carlo
SVD	Singular Value Decomposition
LTI	Linear Time-Invariant
ROI	Region of Interest
PCG	Preconditioned Conjugate Gradient
UAV	Unmanned Aerial Vehicle
NMSE	Normalized Mean Square Error
PDP	Power Delay Profile
PSD	Power Spectral Density
